# Supplementary figures and images for: Intrastriatal injection of Parkinson’s disease intestine and vagus lysates initiates α-synucleinopathy in rat brain
Source: Cell Death Dis. 2023 Jan 5;14(1):4. doi: 10.1038/s41419-022-05531-z (PMC9814765; doi:10.1038/s41419-022-05531-z)

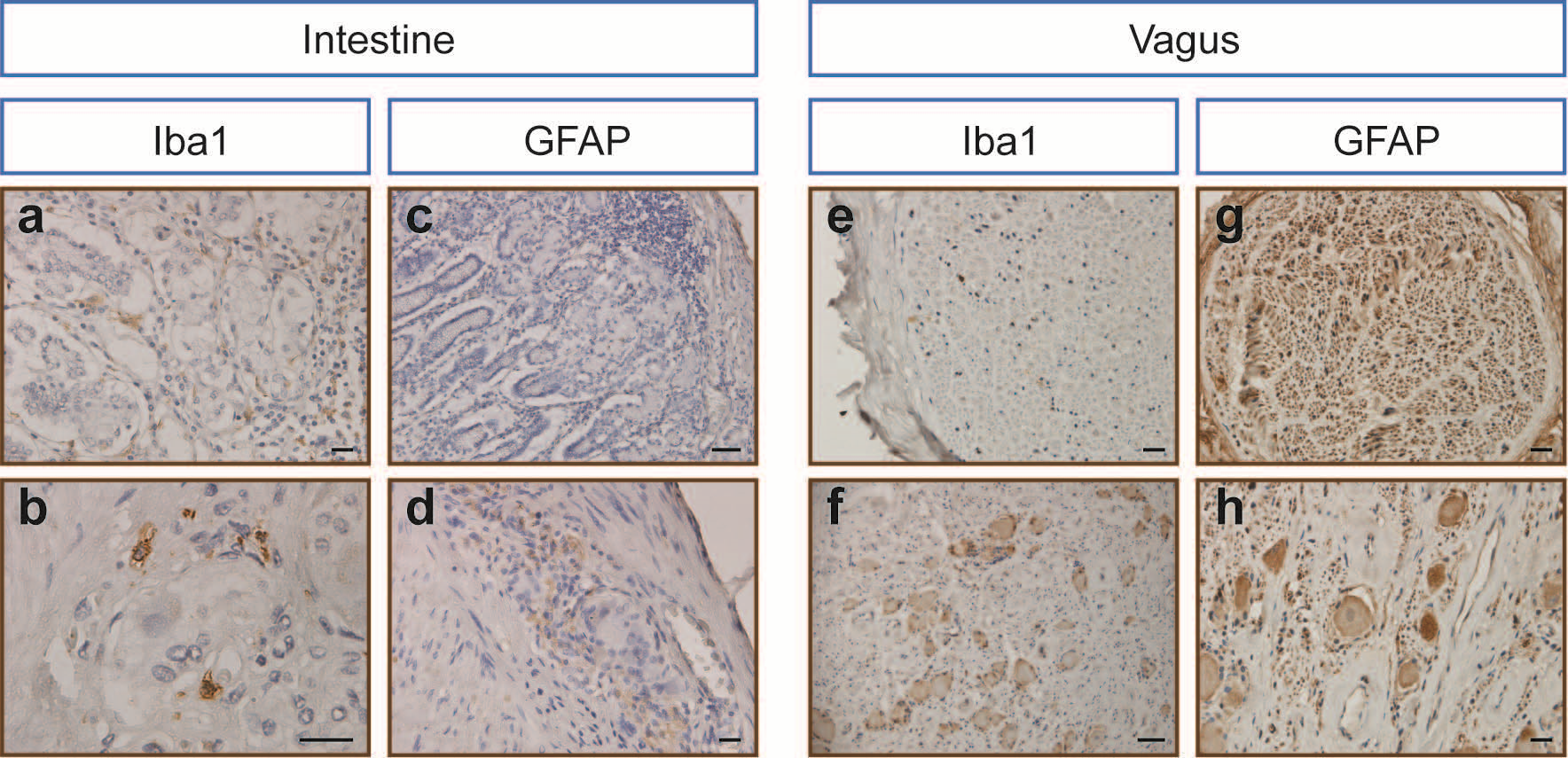

Supplement: Supplementary file 1 — Supplementary Figure 1 [file 41419_2022_5531_MOESM1_ESM.png]

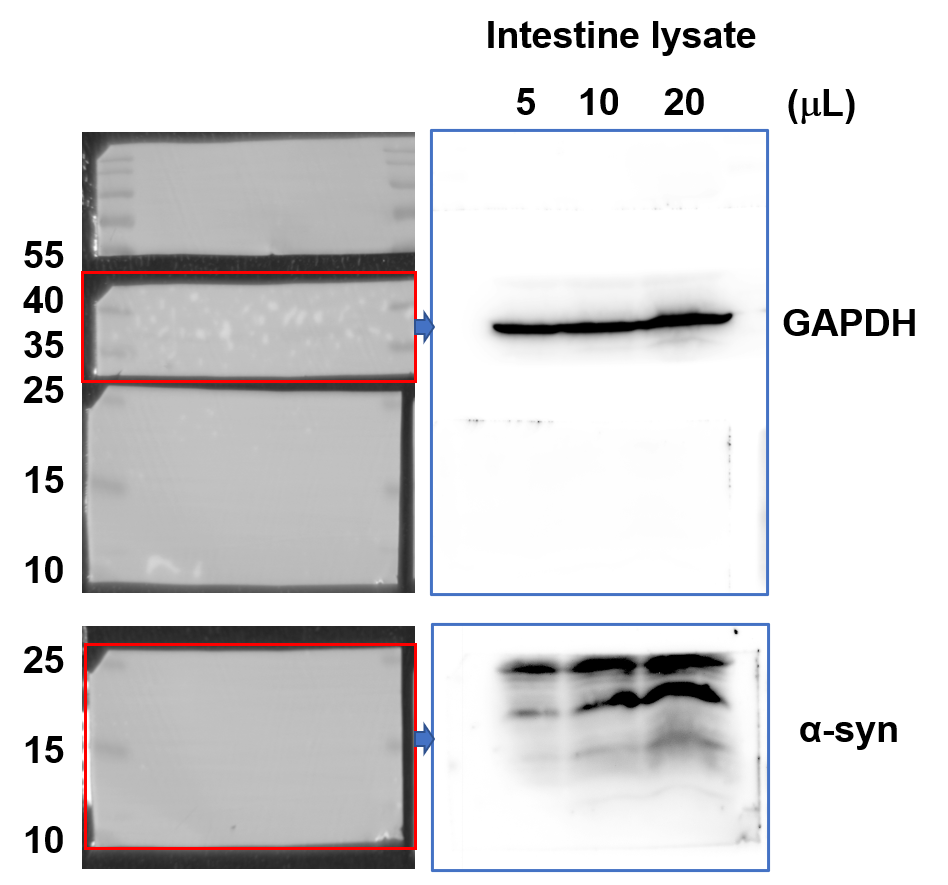

Supplement: Supplementary file 2 — Supplementary Figure 2 [file 41419_2022_5531_MOESM2_ESM.tif]
