# Supplementary material for: Intrastriatal injection of Parkinson’s disease intestine and vagus lysates initiates α-synucleinopathy in rat brain
Source: Cell Death Dis. 2023 Jan 5;14(1):4. doi: 10.1038/s41419-022-05531-z (PMC9814765; doi:10.1038/s41419-022-05531-z)
Supplement: Supplementary file 3 — Supplementary Materials [file 41419_2022_5531_MOESM3_ESM.docx]

**Supplementary Materials**

Title: **Intrastriatal injection of Parkinson’s disease intestine and vagus lysates initiates α-synucleinopathy in rat brain**

Journal name: Cell Death & Disease

Authors: Zhaofei Yang, Ying Wang, Min Wei, Song Li, Congcong Jia, Cheng Cheng, Murad AlNusaif, Jun Zhang, Cong Liu, Weidong Le

Corresponding Author: Address correspondence to Weidong Le (wdle@sibs.ac.cn)

**Inventory of Supplementary Material:**

1. Supplementary Table
2. Supplementary Results
3. Supplementary Figure
4. Full original Western blots

**1. Supplementary Table**

**Table S1.** Antibodies used in this study.

| **Antibody** | **Supplier** | **Catalog Number** | **Staining** | **Working dilution** | **Reactivity** |
| --- | --- | --- | --- | --- | --- |
| Anti-α-synuclein | Santa Cruz Biotechnology | sc-12767 | IHC | 1:50 | human |
| Anti-α-synuclein | BD Transduction Laboratories | 610786 | IHC/IF | 1:1000 | Human, rat |
| Anti- TH | Millipore | AB152 | IF/IHC | 1:1000 | Human, rat |
|  | Aves Labs | TYH | IF | 1:1000 | Human, rat |
| Anti- GFAP | Santa Cruz Biotechnology | sc-33673 | IF | 1:100 | Human, rat |
| Anti- GFAP | Dako | Z0334 | IH | 1:2000 | Human, rat |
| Anti- Iba1 | Wako | 019-19741 | IHC/IF | 1:1000 | Human, rat |

**2. Supplementary Results**

**The expression and distribution of Iba1 and GFAP in the intestine and vagus of a PD patient**

We performed IHC staining on the small intestine of a post-mortem PD patient and found that the distribution of Iba1 is in chains or rings along gland cells in the small intestinal mucosa. Iba1-positive intestinal glial cells could be detected in the myenteric plexus of the small intestine (Figure S1a, b), which was different from that of GFAP. GFAP was less expressed in the mucosa of the small intestine, abundantly enriched in the myenteric plexus, and mainly expressed around the neurons (Figure S1c, d).

In addition, we also detected the vagus nerve derived from the PD patient, showing that there were two main fiber bundles in the ganglion, and Iba1 was mainly distributed in satellite glial cells (Figure S1e, f). We also found that the distribution of GFAP in the vagus nerve was different from that of Iba1. There were various GFAP immunoreactive cells, one shaped as a nested fiber bundle, another expressed in the satellite glial cells around the neuron (Figure S1g, h).

**3. Supplementary Figure**

**
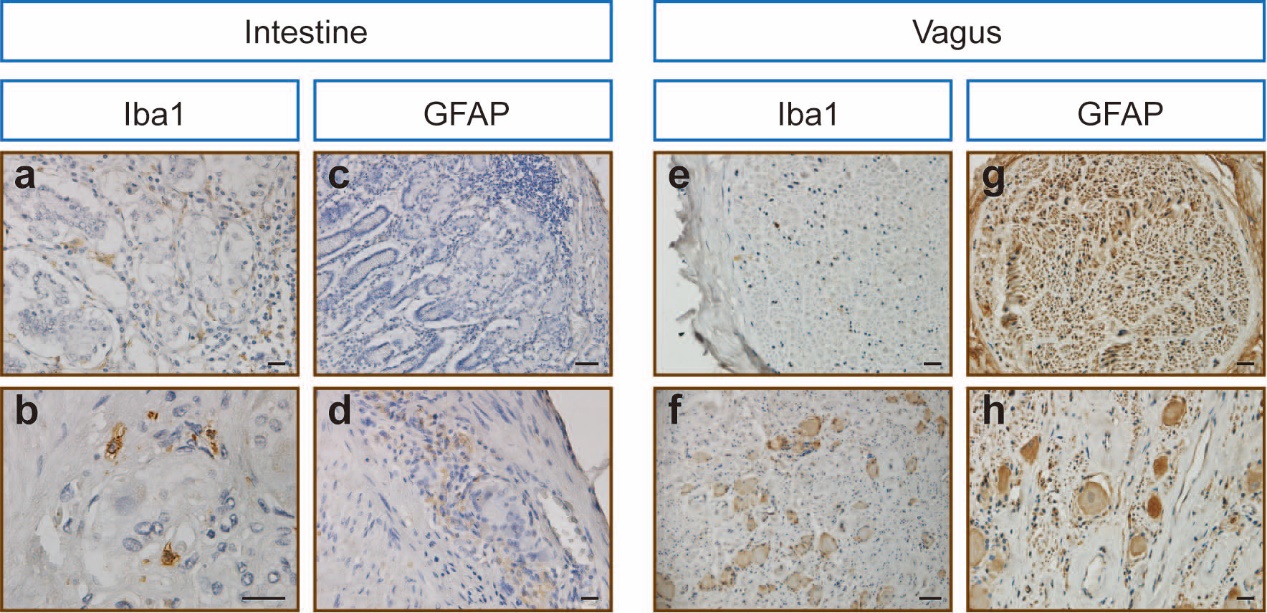
**

**Figure S1.** The expression and distribution of Iba1 and GFAP in the vagus and intestine of a PD patient. Representative images of Iba1 and GFAP expression and distribution in the intestine and vagus of the PD patient are shown. Scale bar: 10 μm **(b)**; 20 μm **(a, d, e, g, h)**; and 50 μm **(c, f)**.

**4. Full original Western blots**


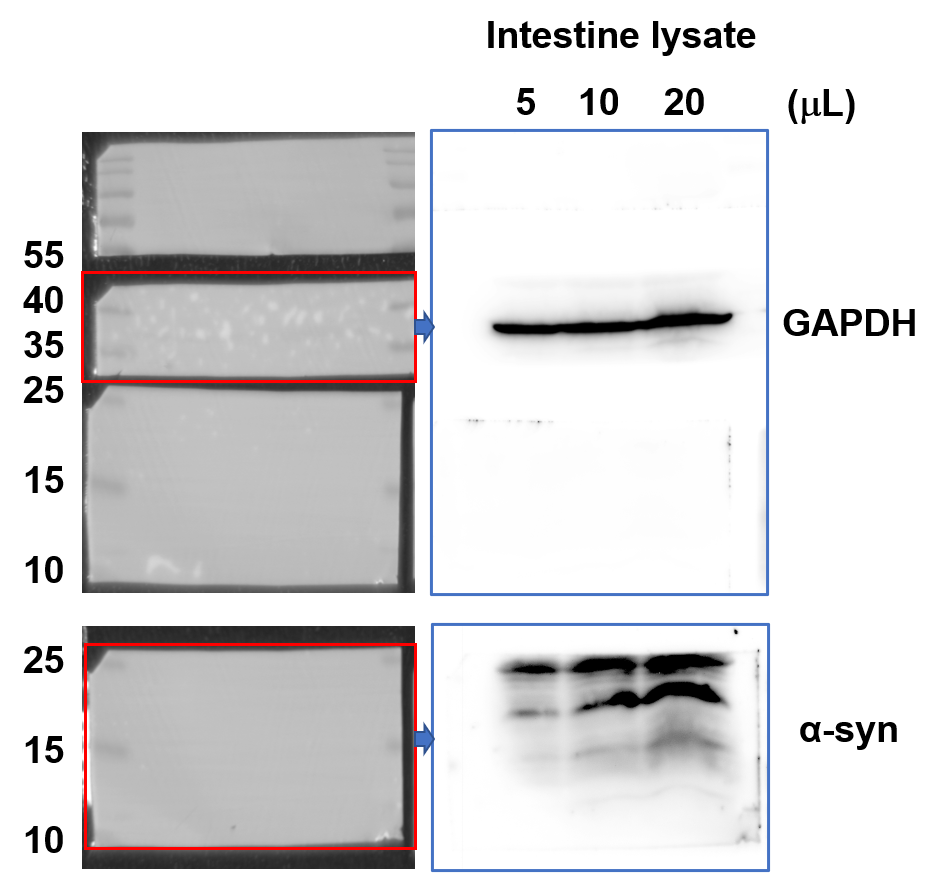


**Figure S2.** The full original Western blots in Figure 2b. Protein markers: ~180, 130, 100, 70, 55, 40, 35, 25, 15, and 10 kDa. The loading quantity of the intestine lysate was 5, 10, and 20 μL, separately.
