# Supplementary material for: Intrastriatal injection of Parkinson’s disease intestine and vagus lysates initiates α-synucleinopathy in rat brain
Source: Cell Death Dis. 2023 Jan 5;14(1):4. doi: 10.1038/s41419-022-05531-z (PMC9814765; doi:10.1038/s41419-022-05531-z)
Supplement: Supplementary file 4 — Manuscript [file 41419_2022_5531_MOESM4_ESM.docx]

Intrastriatal injection of Parkinson’s disease intestine and vagus lysates initiates α-synucleinopathy in rat brain

Zhaofei Yang,^1†^ Ying Wang,^1†^ Min Wei,^1^ Song Li,^1^ Congcong Jia,^1^ Cheng Cheng,^1^ Murad Al-Nusaif,^1^ Jun zhang,^1^ Cong Liu,^2^ and Weidong Le^1, 3^

1 Liaoning Provincial Key Laboratory for Research on the Pathogenic Mechanisms of Neurological Diseases, the First Affiliated Hospital, Dalian Medical University, Dalian 116021, China

2 Interdisciplinary Research Center on Biology and Chemistry, Shanghai Institute of Organic Chemistry, Chinese Academy of Sciences, Shanghai 201210, China

3 Institute of Neurology, Sichuan Academy of Medical Sciences-Sichuan Provincial Hospital, Chengdu 610072, China

**^†^These authors contributed equally to this work.**

Correspondence should be addressed to Prof. Weidong Le, Liaoning Provincial Key Laboratory for Research on the Pathogenic Mechanisms of Neurological Diseases, the First Affiliated Hospital, Dalian Medical University, Dalian 116021, China. Tel./Fax: +86-411-88135850；E-mail: [wdle@sibs.ac.cn](mailto:wdle@sibs.ac.cn)

**Keywords:** Parkinson’s disease; α-synuclein; intestine; vagus nerve; propagation

**Running title**: PD intestine & vagus transmit α-syn pathology

# Abstract

Parkinson’s disease (PD) is characterized by the selective loss of dopaminergic neurons in the midbrain and the pathological accumulation of misfolded α-synuclein (α-syn) in the brain. A growing body of evidence suggests that the formation of misfolded α-syn and aggregation may begin in the peripheral nervous system, specifically the enteric nervous system, and then propagate to the central nervous system via the vagus nerve. However, the PD-like neuropathology induced by the intestine and vagus nerve extracts is rarely investigated. In this work, we injected lysates of the intestine and vagus obtained from a diagnosed PD patient, which contained abnormal α-syn aggregates, into the rat striatum unilaterally. Strikingly, such an injection induced dopaminergic neurodegeneration and α-syn depositions in the striatum, substantia nigra, and other brain regions, including the frontal cortex, somatosensory cortex, hypothalamus, brain stem, and cerebellum. Moreover, significant activation of microglia and the development of astrogliosis were observed in the substantia nigra pars compacta of the injected rats. These findings provide essential information for our understanding of PD pathogenesis, as we established for the first time that the α-syn aggregates in the intestine and vagus of a PD patient were sufficient to induce prion-like propagation of endogenous α-syn pathology in wild-type rats.

# INTRODUCTION

Parkinson’s disease (PD) is the second most common neurodegenerative disease following Alzheimer’s disease, and its occurrence and progression have been extensively studied. However, the pathogenesis of PD is still far from being fully understood. It is generally believed that PD is related to α-synuclein (α-syn) aggregation, neuroinflammation, oxidative stress, and mitochondrial dysfunction in the brain. Non-motor symptoms may appear up to 20 years prior to the occurrence of motor symptoms in PD patients, such as constipation, hyposmia, and sleep disturbances. According to Braak and colleagues, PD pathology may begin in the olfactory bulb (OB) and/or enteric nervous system (ENS), and then spread to the brain via the vagus nerve^1, 2^.

In recent years, the prion-like propagation of α-syn in the gut-brain axis has been proposed and proved in a variety of models^3-6^. The intestine and OB act as gateways of human body to the external environment. Increasing lines of evidence suggest that the α-syn aggregates in ENS and OB are initiated by toxins, pathogens and associated immune or inflammatory reactions, and then spread into connected structures of the central nervous system (CNS) and peripheral nervous system (including ENS and autonomic nervous system) via olfactory tract, sympathetic and parasympathetic nerves^4, 7-10^. The α-syn aggregates can self-propagate, acting as proteinaceous nuclei (“seeds”), and spread the abnormal pathology in a manner reminiscent of infectious prions^11, 12^. Previous reports have demonstrated that intracerebral injection of synthetic α-syn fibrils into different brain regions of mice, rats, and monkeys, including striatum (STR), substantia nigra (SN), hippocampus, and OB, results in the formation of PD-like Lewy pathology in the interconnected brain regions^13-20^. Furthermore, the injection of pathological α-syn species extracted from brains of transgenic mice, PD patients, or patients with other synucleinopathies (such as dementia with Lewy bodies and multiple system atrophy) into wild-type or transgenic animal brains induced propagation of α-syn pathology^21-25^. However, it is still unclear whether α-syn aggregates from human non-brain tissues, such as the intestine and vagus nerve, can trigger α-syn accumulation and propagation in a new host.

In the current study, we extracted the intestine and vagus lysates of a post-mortem PD patient and injected them unilaterally into the left STR of rats, which dramatically induced the accumulation and propagation of α-syn in the brain and affected the dopaminergic neurodegeneration of the nigrostriatal system. Abnormal depositions of aggregated α-syn were found in the ipsilateral frontal cortex (FrC), somatosensory cortex (Ctx), hypothalamus (Hypo-thal), brain stem (BS), and cerebellum (Cere). In addition, it was found that the immune-inflammatory system played a critical role in the communication between the brain and intestine^26, 27^, and microglia and astrocytes were important for the regulation of neuroinflammation^28^. In the present study, increased Ionized calcium-binding adaptor molecule 1 (Iba1) and glial fibrillary acidic protein (GFAP) expression as well as the ratio of reactive Iba1/GFAP-positive cells were found in the SN pars compacta (SNc) of the rats injected with intestine or vagus lysate, which showed prominent microglial activation and astrocytic gliosis, as well as a loss of tyrosine hydroxylase (TH)-positive dopaminergic neurons. The induction of neuropathology and neurologic dysfunction in the inoculated rats provides evidence that the α-syn aggregates present in the intestine and vagus of PD patients act as “seeds”, which can lead to a more precise understanding of the pathological mechanisms of α-syn aggregation and propagation in PD^1, 29^.

# MATERIALS AND METHODS

# Human tissue specimens and patient information

We used the small intestine wall and nodose ganglion (inferior sensory ganglion of the vagus nerve, also known as the vagus nerve/vagus) from a donor with sporadic PD (female, 86 years at death, 14 years disease duration, and ~12 h frozen post-mortem interval) who had nigral Lewy body (LB) pathology on neuropathological examination. The body was obtained from the Body Organ Donation Center of Dalian Medical University. The patient signed the consensual document for body and organ donation (code number: 00303), and the medical history was provided by her next of kin.

## Animals

One-month-old male Sprague Dawley rats (180−220 g) were used in this study. Animals were maintained under specific pathogen-free conditions (temperature, 22 ± 2 °C, 12 h/12 h light-dark cycle) with free access to food and water. Animal care and procedures were carried out in accordance with the Laboratory Animal Care Guidelines approved by the Institutional Animal Care Committee at Dalian Medical University.

## Injection materials

### Intestine lysate

The small intestine wall was dissected from the frozen intestine of a post-mortem PD patient that had been stored at −80 °C. The tissue was immediately refrozen in liquid nitrogen and ground with a mortar and pestle into a fine powder. The frozen powder was mixed with sterile PBS (82 mg powder per 100 μL of PBS). The homogenate was then sonicated 10 times with a handheld probe (SLPe, Branson, USA) at 3 s on/5 s off and centrifugated for 5 min (3000 *g*, 4 °C). The supernatant (lysate) was recovered and stored at −80 °C until injection.

### Vagus lysate

The vagus was dissected from frozen vagus tissue of a post-mortem PD patient that had been stored at −80 °C. The tissue was immediately refrozen in liquid nitrogen and ground with a mortar and pestle into a fine powder. Before vacuum freeze-drying, the frozen powder was mixed with sterile PBS (275 mg powder per 1.375 mL PBS), and the homogenate was stored at −80 °C. For vacuum freeze-drying, vagal/nodose ganglion homogenate samples were placed in the freeze dryer system (7740030, LABCONCO, USA) for vacuum-adsorption, and the homogenate was freeze-dried to 3 g powder at −20 °C for 2 h. Then, the powder was remixed in 30 μL sterile PBS and sonicated at 3 s on/5 s off five times and cleared by centrifugation for 10 min (3000 *g*, 4 °C). Finally, the supernatant (lysate) was recovered and stored at −80 °C until injection.

## Stereotaxic injection

One-month-old male Sprague Dawley rats were anesthetized by isoflurane and stereotaxically injected with intestine lysate (5 µL per brain, n = 8) or vagus lysate (5 µL per brain, n = 4). Control rats received sterile PBS (5 µL per brain, n = 9). Single-needle insertion into the left brain (coordinates: −1.0 mm relative to bregma, 3.5 mm from midline) was used to target the inoculum into STR located at a depth of 4.8 mm below the dura. The material was injected via a Hamilton syringe (80465, Hamilton Company, USA) at a rate of 1 μL per min with the needle in place for 5 min. The needle was left at the injection site for 10 min after the injection.

## Immunostaining

Six months after surgery and injection, the animals were sacrificed under anesthesia and transcardially perfused with cold PBS. The brain was removed for histological studies and postfixed in 4% paraformaldehyde for 24 h before being dehydrated in 15% and 30% sucrose at 4 °C for 24 h.

For immunohistochemical (IHC) staining, a rabbit (PV-9001, ZSGB-BIO Company, Beijing, China) or mouse (PV-9002, ZSGB-BIO Company, Beijing, China) two-step detection kit was used, as described previously^30^. A series of 40 µm thick slides were incubated in Solution A for 10 min. After rinsing with PBS three times, blocking solution (10% normal goat serum, 0.2% Triton-X 100, and 0.05% NaN_3_ in PBS) was applied overnight at 4 °C. Detailed information on primary antibodies and working dilutions are listed in Table S1. Solutions B and C were used according to the manufacturer’s instructions and after subsequent exposure to diaminobenzidine (ZLI-9019, ZSGB-BIO Company, China) for 5 min. After rinsing with PBS, the sections were dehydrated through a graded ethanol series. Finally, IHC staining images were visualized directly by DP80 CCD brightfield microscopy (Olympus, Japan). The outlines of the SNc, ventral tegmental area (VTA), dorsal STR, FrC, Ctx, Hypo-thal, BS, and Cere were delimited according to anatomical landmarks^31^.

For immunofluorescence (IF) staining, 40 µm thick sections were incubated overnight at 4 °C in blocking solution (10% normal goat serum, 0.2% Triton-X 100, and 0.05% NaN_3_ in PBS). Detailed information on primary antibodies and working dilutions are listed in Table S1. For the immunostaining of GFAP, the blocking steps were performed after antigen retrieval (citrate buffer containing 3 g trisodium citrate and 0.4 g citrate diluted in 1 L distilled water, pH 6.0). Finally, images were directly visualized and captured using a confocal microscope (A1 confocal, Nikon Instruments Co., Ltd.) and a DP80 CCD brightfield microscope (Olympus, Japan). The outline of the SNc was defined according to anatomical landmarks.

## Image analysis

TH^-^positive cells in the SNc and VTA were calculated every three sections from −4.44 to −6.72 mm Bregma (The Rat Brain in Stereotaxic Coordinates by George Paxinos & Charles Watson, 5th Edition) at 10× magnification by an observer who was blind to the genotype, and data were collected from 11 slices per animal. The IHC intensity was analyzed using ImageJ software, and the data were collected from 2−3 slices per animal.

## High-performance liquid chromatography

The rat brain was rapidly dissected, and the whole STR in the left brain was isolated. For high-performance liquid chromatography (HPLC) analysis, the STR specimen was weighed, 0.2 M perchloric acid was added, including 100 µM EDTA-2Na (5 µL per mg of tissue), and then it was sonicated on ice with 2 s on/8 s off 2-4 times. The homogenate was kept in an ice bath for 30 min. After vortexing at 17,000 *g* for 15 min at 4 °C, the supernatant was modified to pH 3.0 using 1 M sodium acetate. The samples were filtered through a 0.45 μm syringe filter and analyzed by HPLC (HTEC-500, EICOM, Japan) through the electrochemical detection method. Different dopamine (DA) standard concentrations were detected to plot a standard curve for data analysis.

## Statistical analysis

Data were expressed as means ± SEM and were analyzed using GraphPad Prism software (Version 7.0). Statistical comparisons were performed using Student’s two-tailed unpaired t-test, as indicated in the figure legends, and *p* < 0.05 was considered significant. All experiments were repeated at least three times.

# RESULTS

## Human α-syn pathology in the rat brain injected with intestine or vagus lysate derived from a pathologically confirmed PD case

The experimental rats received a single unilateral stereotaxic inoculation of intestine lysate (24 pg/µL, α-syn), vagus lysate (20 pg/µL, α-syn), or PBS (vehicle control) above the left STR. The animals were euthanized at six months following inoculations, and their brains were processed for IHC and IF staining. The experimental timeline and methods are shown in Figure 1.

Before inoculating the rats with intestine or vagus lysate from the PD case, we sought to determine whether the intestine and vagus of the PD patient contained α-syn aggregates (“seeds”) that could transmit disease to wild-type rats. IHC staining of the intestine showed α-syn-positive cells in the mucosa and α-syn deposits in the submucosal nerve plexus. The vagus nerve used in this study was the nodose ganglion (inferior ganglion of the vagus nerve), the larger sensory ganglion of the vagus. The IHC staining revealed two main nerve bundles in the ganglion, and there were various α-syn immunoreactive cells. In one type of fiber bundle, α-syn immunoreactive cells were distributed in a nested pattern, while the α-syn-positive aggregation in another fiber bundle was punctate particles concentrated in the cytoplasm (Figure 2a). We also demonstrated that the intestine lysate contained extractable human α-syn species using Western blotting, and the expression levels of α-syn species were upregulated with the increase in the detection volume of the intestine lysate (5, 10, and 20 μL) in a dose-dependent manner (Figure 2b). The original Western blots were present in Figure S2.

Furthermore, we found that intracerebral injection with intestine or vagus lysate from the PD patient resulted in the formation of intracellular LB/Lewy neurite-like inclusions in the ipsilateral (left) and contralateral (right) SN and STR of the recipient animals using an antibody (Syn211) that specifically recognizes amino acid residues 121−125 in both soluble and pathological (LB-linked) α-syn of human origin. The accumulation of α-syn aggregates exhibited different phenotypes, including small, diffuse cytoplasmic inclusions and dense, perinuclear LB-like α-syn inclusions. No human α-syn was detected in the vehicle controls (Figure 2c). Taken together, these findings demonstrate that exogenous human α-syn species were taken up within the host neurons in intestine/vagus lysate-injected rats.

## Unilateral intestine or vagus lysate injection resulted in bilateral accumulation of α-syn aggregates in the SN and STR

To further analyze the pathological changes induced by human α-syn “seeds”, we examined both exogenous and endogenous α-syn expression and distribution using an antibody that recognizes pathogenic human and rodent α-syn. In comparison to the vehicle control, rats that were unilaterally inoculated with intestine or vagus lysate developed significant α-syn pathology including intracellular LB/Lewy neurite-like inclusions in the bilateral SN, VTA (Figure 3a), and STR (Figure 3b), implying that human α-syn “seeds” in the intestine and vagus can induce endogenous α-syn intraneuronal propagation and aggregation.

**Unilateral intestine or vagus lysate injection caused nigrostriatal dopaminergic neurodegeneration in the bilateral brain**

The mesodiencephalic dopaminergic system, at the levels of both SNc and VTA, was assessed by stereological cell counts of dopaminergic TH-positive neurons in the bilateral brain. The optical densitometry of striatal dopaminergic TH-positive fibers was also detected. By six months, intestine or vagus lysate-injected rats exhibited nigrostriatal degeneration of SNc dopaminergic neuron (intestine, *p* = 0.0053, n = 3; vagus, *p* = 0.0082, n = 3) and striatal TH-positive fibers (intestine, *p* = 0.0376, n = 4), compared to vehicle controls (Figure 4a, b). However, no evidence of neurodegeneration in the VTA was observed (Figure 4a). This finding suggests that the human α-syn “seeds” in the intestine and vagus could induce α-syn aggregates accumulation and transmission in the mesodiencephalic dopaminergic system of rats, which might contribute to nigrostriatal neurodegeneration.

Thus, HPLC analysis was also utilized to assess the levels of striatal DA and its metabolites in the rats injected with intestine lysate after six months, and we directly compared them to the age-matched vehicle controls. The expression levels of DA and homovanillic acid (HVA) decreased significantly after the inoculation (DA, −20%, *p* = 0.0238, n = 4; HVA, −17%, *p* = 0.0439, n = 4), while no significant alteration was observed in 3,4-dihydroxyphenylacetic acid (DOPAC) concentration (Figure 4c). We further analyzed the DOPAC/DA and HVA/DA ratios, which did not change, indicating that the decreased DA levels were due to a decrease in DA production.

## Unilateral intestine or vagus lysate injection led to widespread α-syn aggregation throughout the bilateral brain

To determine whether human α-syn “seeds” in the intestine or vagus of a PD patient could cause the pathological conversion of endogenous α-syn in other brain regions, we also examined α-syn pathology in the bilateral brain regions using an antibody that recognizes pathogenic human and rodent α-syn. In addition to SN and STR, prominent intracellular α-syn aggregates were visible in several other brain regions, including FrC, Ctx, Hypo-thal, BS, and Cere, which project direct or indirect innervation to the STR. In contrast, no α-syn pathology was detected in age-matched vehicle controls (Figure 5).

## Unilateral intestine or vagus lysate injection induced microglia activation and astrogliosis in bilateral SNc

Several recent reports indicate that glial cells, especially microglia, play a direct role in the transmission of α-syn pathology in PD^32-36^. To explore the effect of intraneuronal α-syn aggregate accumulation on the surrounding glial cells, we examined the active status of microglia and astrocyte in the bilateral SNc of intestine or vagus lysate-injected rats compared to the vehicle controls. Reactive Iba1-positive cells were detected in both ipsilateral and contralateral SNc of the rats, but more were identified in intestine or vagus lysate-injected rats, which reached statistical significance (intestine, *p* = 0.015, n = 3; vagus, *p* = 0.0443, n = 3). The ratio of reactive Iba1-positive cells was 83% in intestine lysate-injected rats, 70% in vagus lysate-injected rats, and around 36% in PBS-injected rats (Figure 6a).

Interestingly, a similar augmentation of reactive GFAP-positive cells was observed in bilateral SNc of intestine or vagus lysate-injected rats (intestine, *p* = 0.0009, n = 3; vagus, *p* = 0.0403, n = 3). The ratio of reactive GFAP-positive cells reached 61% in intestine lysate-injected rats, 55% in vagus lysate-injected rats, and around 26% in PBS-injected rats (Figure 6b). In addition, we also explored Iba1 and GFAP expression in the intestine and vagus of the PD patient, as shown in Figure S1. Taken together, these findings demonstrate that intestine or vagus lysate-injected rats exhibited microglial activation and astrogliosis in the SNc, concomitant with dopaminergic neurodegeneration.

# DISCUSSION

In the present study, we investigated whether α-syn “seeds” from non-brain tissue can trigger α-syn accumulation and propagation in a new host. Intrastriatal injection of either intestine or vagus lysate derived from a post-mortem PD patient, instead of well-defined α-syn assemblies (oligomers, ribbons, or fibrils), for which it has been found that distinct α-syn strains display differential seeding capacities in the rat brain^37^. We demonstrate that the α-syn aggregates contained in the intestine and vagus of the PD patient are pathogenic, which can induce a PD-like pathological process, including nigrostriatal dopaminergic neurodegeneration accompanied by reactive microglia and astrogliosis in SNc, and intraneuronal α-syn aggregates accumulate and transmit through the bilateral brain.

Recent reports suggest that pathological α-syn aggregates in the ENS/vagus nerve can initiate α-syn deposition and propagation and retrogradely spread to the brain by directly injecting recombinant α-syn fibrils to vagus nerve/duodenum muscle layers of wild-type/transgenic rodent models^38, 39^. Moreover, Wang *et al.* demonstrated that the local injection of α-syn preformed fibrils in sympathetic ganglia induces progressive α-syn pathology in the autonomic nervous system in M83 transgenic mice^9^. More support for this finding was provided by Kim et al., who injected recombinant mouse α-syn preformed fibrils into the pylorus and duodenum muscle layers of mice. By 10 months after injection, the pathologic α-syn had spread to the mouse dorsal motor nucleus, SNc, hippocampus, STR, FrC, and olfactory bulb. Interestingly, the spread of pathologic α-syn from the gut to the brain and the PD-like phenotypes did not occur in truncal vagotomized mice or α-syn knockout mice^39^. The above studies and our research demonstrate that the peripheral nervous system including ENS, autonomic nervous system, and vagus nerve play essential roles in the initiation and progression of PD pathology.

Several mechanisms involved in α-syn transneuronal propagation have been reported, including exocytosis (exosomes, extracellular vesicles, non-classical exocytosis, or exophagy), endocytosis, axonal transport, tunneling nanotubes, and direct penetration^33, 40, 41^. Mounting evidence suggests that activated microglia enhance the transmission of α-syn in PD. A recent study revealed that plasma exosomes derived from PD patients carry pathological α-syn and target microglia preferentially, and their intrastriatal injection resulted in the transmission of exosomal α-syn from microglia to neurons following microglia activation^35^. In this study, we also found microglia activation and astrogliosis in bilateral SNc of rats, accompanied by depositions of α-syn aggregation and loss of TH-positive neurons (Figure 6). In addition, we performed immunohistochemical staining on the intestine and nodose ganglion (containing the vagus nerve) of the PD autopsy patient and found that the expressions of GFAP and Iba1 were distributed differently in the vagus nerve and intestine (Figure S1). To explore the underlying mechanisms and potential therapeutic targets of α-syn aggregation and propagation, further studies are needed in wild-type rats by injecting vagus and intestine lysates from age-matched non-PD human subjects as control.

The current study found that intrastriatal injection of intestine or vagus lysate derived from a PD patient can induce α-syn transmission and nigrostriatal neurodegeneration in rats six months post-injection (Figure 4). Many scientists have recently explored whether transferred α-syn can propagate and induce neurological impairment or disease in a new host using α-syn species from PD patients^11, 21, 25, 42-45^. For example, the study by Recasens *et al.* indicated that intracerebral injection of LB extracts from PD patients caused α-syn pathology and nigrostriatal neurodegeneration in the brain of wild-type mice and macaques^21^. Thomzig *et al.* suggested that intracerebral injection of brain and stomach wall homogenates from PD patients initiates α-syn aggregation, but it is not accompanied by apparent motor impairments in TgM83^+/−^ mice up to 612 days post-injection^11^. Most of these results are consistent with our present research. In contrast, Prusiner *et al.* found that transmission of homogenates from PD brains did not stimulate pathological α-syn inclusions or clinical symptoms in transgenic TgM83^+/−^ mice expressing human A53T mutated α-syn “>360” days post-injection^46^. These studies indicate the effects of recipient, species barrier, inoculation composition (different conformational strains of α-syn), injection site, and the observation time post-injection are crucial factors that cannot be ignored, as each of these variables may result in distinct neuropathology and motor symptoms.

To the best of our current knowledge, this is the first report on the stimulation of pathological α-syn aggregation and transmission in the brain of bioassay animals by inoculation with intestine or vagus lysate from a PD patient. The present study substantiated that transmitted α-syn “seeds” from non-CNS tissues can propagate in new mammalian recipients. The effort to explore the susceptible nerve cell types and α-syn transmission mechanisms among cells in human CNS, ENS, and peripheral nervous systems is important for understanding the degenerative process of sporadic PD. Moreover, potential safeguards need to be investigated in the future.

## DATA AVAILABILITY

Any data not published within the article are available from the corresponding author upon reasonable request.

**ABBREVIATIONS**

α-syn, α-synuclein; BS, brain stem; Cere, cerebellum; Ctx, somatosensory cortex; DA, dopamine; DOPAC, 3,4-dihydroxyphenylacetic acid; ENS, enteric nervous system; FrC, frontal cortex; GFAP, glial fibrillary acidic protein; HPLC, high-performance liquid chromatography; HVA, homovanillic acid; Hypo-thal, hypothalamus; Iba1, ionized calcium-binding adaptor molecule 1; IF, immunofluorescence; IHC, immunohistochemistry; SN, substantia nigra; SNc, substantia nigra pars compacta; STR, striatum; TH, tyrosine hydroxylase; VTA, ventral tegmental area; L, ipsilateral left brain; R, contralateral right brain.

# ACKNOWLEDGEMENTS

We are cordially grateful to the PD patient for the body donation and her family members’ support and cooperation, to Lei Sun, Fei Xu, Hongjin Sui and Shengming Yin for the participation in the body donation, to Jinyong Peng and Lianhong Yin for assistance in preparing vagus lysate, and to all members in our laboratory for caring, help and advice.

**AUTHOR CONTRIBUTIONS**

LW designed the experiments. YZF and WY contributed to injection materials and stereotaxic injection and data analysis. WM, LS, JCC, and CC contributed to imaging experiments and data analysis. ZJ provided technical and material support. YZF, WY, AlNusaif M, LC and LW wrote and edited the manuscript. All authors read and approved the final manuscript.

# FUNDING

This work was supported by the National Basic Research Program of China (81771521 and 82101513), the National Key R&D Program (2016YFC1306600), and Guangdong Provincial Key R&D Program (2018B030337001).

**ETHICAL APPROVAL**

Animal care and procedures were carried out in accordance with the Laboratory Animal Care Guidelines approved by the Institutional Animal Care Committee at Dalian Medical University. The protocol was approved by the Institutional Animal Care Committee at Dalian Medical University.

# COMPETING INTERESTS

The authors report no competing interests.

# ADDITIONAL INFORMATION

Supplementary material is available at Cell Death & Disease online.

**REFERENCES**

1. Del Tredici K, Braak H. Review: Sporadic Parkinson’s disease: development and distribution of α-synuclein pathology. Neuropathol Appl Neurobiol. 2016;42:33-50.

2. Braak H, Del Tredici K. Neuropathological Staging of Brain Pathology in Sporadic Parkinson’s disease: Separating the Wheat from the Chaff. J Parkinsons Dis. 2017;7:S71-S85.

3. Klingelhoefer L, Reichmann H. Pathogenesis of Parkinson disease--the gut-brain axis and environmental factors. Nat Rev Neurol. 2015;11:625-636.

4. Perez-Pardo P, Kliest T, Dodiya HB, Broersen LM, Garssen J, Keshavarzian A, et al. The gut-brain axis in Parkinson’s disease: Possibilities for food-based therapies. Eur J Pharmacol. 2017;817:86-95.

5. Chen Y, Shao Q, Yuan YH, Chen NH. Prion-like propagation of α-synuclein in the gut-brain axis. Brain Res bull. 2018;140:341-346.

6. Chetty D, Abrahams S, van Coller R, Carr J, Kenyon C, Bardien S. Movement of prion-like α-synuclein along the gut-brain axis in Parkinson’s disease: A potential target of curcumin treatment. Eur J Neurosci. 2021;54:4695-4711.

7. Van Den Berge N, Ferreira N, Gram H, Mikkelsen TW, Alstrup AKO, Casadei N, et al. Evidence for bidirectional and trans-synaptic parasympathetic and sympathetic propagation of alpha-synuclein in rats. Acta Neuropathol. 2019;138:535-550.

8. Van Den Berge N, Ferreira N, Mikkelsen TW, Alstrup AKO, Tamgüney G, Karlsson P, et al. Ageing promotes pathological alpha-synuclein propagation and autonomic dysfunction in wild-type rats. Brain. 2021;144:1853-1868.

9. Wang XJ, Ma MM, Zhou LB, Jiang XY, Hao MM, Teng RKF, et al. Autonomic ganglionic injection of α-synuclein fibrils as a model of pure autonomic failure α-synucleinopathy. Nat Commun. 2020;11:934.

10. Pan-Montojo F, Schwarz M, Winkler C, Arnhold M, O’Sullivan GA, Pal A, et al. Environmental toxins trigger PD-like progression via increased alpha-synuclein release from enteric neurons in mice. Sci Rep. 2012;2:898.

11. Thomzig A, Wagenführ K, Pinder P, Joncic M, Schulz-Schaeffer WJ, Beekes M. Transmissible α-synuclein seeding activity in brain and stomach of patients with Parkinson’s disease. Acta Neuropathol. 2021;141:861-879.

12. Soto C, Pritzkow S. Protein misfolding, aggregation, and conformational strains in neurodegenerative diseases. Nat Neurosci. 2018;21:1332-1340.

13. Luk KC, Kehm V, Carroll J, Zhang B, O’Brien P, Trojanowski JQ, et al. Pathological α-synuclein transmission initiates Parkinson-like neurodegeneration in nontransgenic mice. Science. 2012;338:949-953.

14. Abdelmotilib H, Maltbie T, Delic V, Liu Z, Hu X, Fraser KB, et al. α-Synuclein fibril-induced inclusion spread in rats and mice correlates with dopaminergic Neurodegeneration. Neurobiol Dise. 2017;105:84-98.

15. Masuda-Suzukake M, Nonaka T, Hosokawa M, Kubo M, Shimozawa A, Akiyama H, et al. Pathological alpha-synuclein propagates through neural networks. Acta Neuropathol Commun. 2014;2:88.

16. Rey NL, Petit GH, Bousset L, Melki R, Brundin P. Transfer of human α-synuclein from the olfactory bulb to interconnected brain regions in mice. Acta Neuropathol. 2013;126:555-573.

17. Sacino AN, Brooks M, McKinney AB, Thomas MA, Shaw G, Golde TE, et al. Brain injection of α-synuclein induces multiple proteinopathies, gliosis, and a neuronal injury marker. J Neurosci. 2014;34:12368-12378.

18. Shimozawa A, Ono M, Takahara D, Tarutani A, Imura S, Masuda-Suzukake M, et al. Propagation of pathological α-synuclein in marmoset brain. Acta Neuropathol Commun. 2017;5:12.

19. Paumier KL, Luk KC, Manfredsson FP, Kanaan NM, Lipton JW, Collier TJ, et al. Intrastriatal injection of pre-formed mouse α-synuclein fibrils into rats triggers α-synuclein pathology and bilateral nigrostriatal degeneration. Neurobiol Dis. 2015;82:185-199.

20. Uemura N, Yagi H, Uemura MT, Hatanaka Y, Yamakado H, Takahashi R. Inoculation of α-synuclein preformed fibrils into the mouse gastrointestinal tract induces Lewy body-like aggregates in the brainstem via the vagus nerve. Mol Neurodegener. 2018;13:21.

21. Recasens A, Dehay B, Bové J, Carballo-Carbajal I, Dovero S, Pérez-Villalba A, et al. Lewy body extracts from Parkinson disease brains trigger α-synuclein pathology and neurodegeneration in mice and monkeys. Ann Neurol. 2014;75:351-362.

22. Bernis ME, Babila JT, Breid S, Wüsten KA, Wüllner U, Tamgüney G. Prion-like propagation of human brain-derived alpha-synuclein in transgenic mice expressing human wild-type alpha-synuclein. Acta Neuropathol Commun. 2015;3:75.

23. Watts JC, Giles K, Oehler A, Middleton L, Dexter DT, Gentleman SM, et al. Transmission of multiple system atrophy prions to transgenic mice. Proc Natl Acad Sci U S A. 2013;110:19555-19560.

24. Luk KC, Kehm VM, Zhang B, O’Brien P, Trojanowski JQ, Lee VM. Intracerebral inoculation of pathological α-synuclein initiates a rapidly progressive neurodegenerative α-synucleinopathy in mice. J Exp Med. 2012;209:975-986.

25. Kawakami I, Motoda A, Hashimoto M, Shimozawa A, Masuda-Suzukake M, Ohtani R, et al. Progression of phosphorylated α-synuclein in Macaca fuscata. Brain Pathol. 2021;31:e12952.

26. Sampson TR, Debelius JW, Thron T, Janssen S, Shastri GG, Ilhan ZE, et al. Gut Microbiota Regulate Motor Deficits and Neuroinflammation in a Model of Parkinson’s Disease. Cell. 2016;167:1469-1480.e12.

27. Braak H, Ghebremedhin E, Rüb U, Bratzke H, Del Tredici K. Stages in the development of Parkinson’s disease-related pathology. Cell Tissue Res. 2004;318:121-134.

28. Wang Q, Song S, Jiang L, Hong JS. Interplay among norepinephrine, NOX2, and neuroinflammation: key players in Parkinson’s disease and prime targets for therapies. Ageing Neur Dis. 2021;1:6.

29. Dehay B, Bourdenx M, Gorry P, Przedborski S, Vila M, Hunot S, et al. Targeting α-synuclein for treatment of Parkinson’s disease: mechanistic and therapeutic considerations. Lancet Neurol. 2015;14:855-866.

30. Merz H, Malisius R, Mannweiler S, Zhou R, Hartmann W, Orscheschek K, et al. ImmunoMax. A maximized immunohistochemical method for the retrieval and enhancement of hidden antigens. Laboratory Invest 1995;73:149-156.

31. Bakker R, Tiesinga P, Kötter R. The Scalable Brain Atlas: Instant Web-Based Access to Public Brain Atlases and Related Content. Neuroinformatics. 2015;13:353-366.

32. Sorrentino ZA, Giasson BI, Chakrabarty P. α-Synuclein and astrocytes: tracing the pathways from homeostasis to neurodegeneration in Lewy body disease. Acta Neuropathol. 2019;138:1-21.

33. Xia Y, Zhang G, Han C, Ma K, Guo X, Wan F, et al. Microglia as modulators of exosomal alpha-synuclein transmission. Cell Death Dis. 2019;10:174.

34. Guo M, Wang J, Zhao Y, Feng Y, Han S, Dong Q, et al. Microglial exosomes facilitate α-synuclein transmission in Parkinson’s disease. Brain. 2020;143:1476-1497.

35. Xia Y, Zhang G, Kou L, Yin S, Han C, Hu J, et al. Reactive microglia enhance the transmission of exosomal α-synuclein via toll-like receptor 2. Brain. 2021;144:2024-2037.

36. Zheng T, Zhang Z. Activated microglia facilitate the transmission of α-synuclein in Parkinson’s disease. Neurochem Int. 2021;148:105094.

37. Peelaerts W, Bousset L, Van der Perren A, Moskalyuk A, Pulizzi R, Giugliano M, et al. α-Synuclein strains cause distinct synucleinopathies after local and systemic administration. Nature. 2015;522:340-344.

38. Ulusoy A, Rusconi R, Pérez-Revuelta BI, Musgrove RE, Helwig M, Winzen-Reichert B, et al. Caudo-rostral brain spreading of α-synuclein through vagal connections. EMBO Mol Med. 2013;5:1119-1127.

39. Kim S, Kwon SH, Kam TI, Panicker N, Karuppagounder SS, Lee S, et al. Transneuronal Propagation of Pathologic α-Synuclein from the Gut to the Brain Models Parkinson’s Disease. Neuron. 2019;103:627-641.e7.

40. Freundt EC, Maynard N, Clancy EK, Roy S, Bousset L, Sourigues Y, et al. Neuron-to-neuron transmission of α-synuclein fibrils through axonal transport. Ann Neurol. 2012;72:517-524.

41. Vargas JY, Grudina C, Zurzolo C. The prion-like spreading of α-synuclein: From in vitro to in vivo models of Parkinson’s disease. Ageing Res Rev. 2019;50:89-101.

42. Holmqvist S, Chutna O, Bousset L, Aldrin-Kirk P, Li W, Björklund T, et al. Direct evidence of Parkinson pathology spread from the gastrointestinal tract to the brain in rats. Acta Neuropathol. 2014;128:805-820.

43. Jones DR, Delenclos M, Baine AT, DeTure M, Murray ME, Dickson DW, et al. Transmission of Soluble and Insoluble α-Synuclein to Mice. J Neuropathol Exp Neurol. 2015;74:1158-1169.

44. Dhillon JS, Trejo-Lopez JA, Riffe C, Levites Y, Sacino AN, Borchelt DR, et al. Comparative analyses of the in vivo induction and transmission of α-synuclein pathology in transgenic mice by MSA brain lysate and recombinant α-synuclein fibrils. Acta Neuropathol Commun. 2019;7:80.

45. Arotcarena ML, Dovero S, Prigent A, Bourdenx M, Camus S, Porras G, et al. Bidirectional gut-to-brain and brain-to-gut propagation of synucleinopathy in non-human primates. Brain. 2020;143:1462-1475.

46. Prusiner SB, Woerman AL, Mordes DA, Watts JC, Rampersaud R, Berry DB, et al. Evidence for α-synuclein prions causing multiple system atrophy in humans with parkinsonism. Proc Natl Acad Sci U S A. 2015;112:E5308- E5317.

# Figure legends

**Figure 1.** Experimental timeline. One-month-old male Sprague Dawley rats received unilateral intrastriatal injection of PBS (vehicle control), intestine lysate, or vagus lysate derived from a post-mortem PD patient. The animals injected with PBS (n = 9), intestine lysate (n = 8), or vagus lysate (n = 4) were sacrificed. Paraformaldehyde-fixed tissue sections were processed for IHC and IF to assess both human and rat α-syn accumulation in brain; TH-positive neuron/intensity in SNc, VTA, and STR; and Iba1- and GFAP-positive cells in SNc. HPLC analysis was conducted in the intestine lysate group to assess striatal dopamine levels six months post-injection. DA, dopamine; DOPAC, 3,4-dihydroxyphenylacetic acid; GFAP, glial fibrillary acidic protein; HPLC, high-performance liquid chromatography; HVA, homovanillic acid; Iba1, ionized calcium-binding adaptor molecule 1; IF, immunofluorescence; IHC, immunohistochemistry; SNc, substantia nigra pars compacta; STR, striatum; TH, tyrosine hydroxylase; VTA, ventral tegmental area.

**Figure 2.** Human α-syn expression in the rats injected with intestine or vagus lysate derived from a PD patient. (**a**) Immunohistochemistry staining shows human α-syn aggregations in the mucosa and submucosal plexus of intestine (left), Schwann cells, and nodose ganglion (right) of the post-mortem PD patient. (Scale bars, 20 μm; high-magnification, 10 μm). (**b**) The extracted intestine lysate contains α-syn aggregates, and the expression level of α-syn increased in a volume-dependent manner. The loading quantity of intestine lysate was 5, 10, and 20 μL, separately. (**c**) The human-derived α-syn accumulation in the SN and STR of rats injected with PBS, intestine lysate, or vagus lysate. Scale bars, 20 μm. L, ipsilateral left brain; R, contralateral right brain; SN, substantia nigra; STR, striatum.

**Figure 3.** Increased somatic accumulation of α-syn in the rats injected with intestine or vagus lysate. α-syn is immunoreactive in the neurons and neuritis of host midbrain SN (**a**) and STR (**b**) in the rats intrastriatally injected with PBS, intestine lysate, or vagus lysate derived from a post-mortem PD patient. Arrows point to dopaminergic neurons containing a large number of pigmented granules. Scale bars, 200 μm; high-magnification, 20 μm. L, ipsilateral left brain; R, contralateral right brain.

**Figure 4.** The mesodiencephalic dopaminergic neuronal loss and profound fiber pathology in the rats injected with intestine or vagus lysate. (**a**) Representative coronal brain sections of TH immunostaining in the SNc and VTA of rats injected with PBS, intestine lysate, or vagus lysate. The TH-positive dopaminergic neurons of SNc were significantly impaired in the rats with intestine or vagus lysate injection compared to vehicle control. Unpaired t-test, ** p = 0.0053 (Intestine), ** p = 0.0082 (Vagus). Scale bars, 200 μm. (**b**) Representative coronal brain sections of TH immunostaining in the STR of rats injected with PBS, intestine lysate, or vagus lysate. The TH intensity in STR was decreased significantly in the rats with intestine or vagus lysate injection. Unpaired t-test, * p = 0.0376 (Intestine). Scale bars, 100 μm. (**c**) HPLC results indicate that the levels of DA and its HVA metabolites significantly decreased in the rats with intestine lysate injection, compared to vehicle control. The changes in DOPAC levels and the ratios of HVA/DA and DOPAC/DA were not obvious. Unpaired t-test, * p = 0.0238 (DA), * p = 0.0439 (HVA). DA, dopamine; DOPAC, 3,4-dihydroxyphenylacetic acid; HVA, homovanillic acid; SNc, substantia nigra pars compacta; STR, striatum; TH, tyrosine hydroxylase; VTA, ventral tegmental area.

**Figure 5.** Cell-cell transmission of α-syn aggregation in bilateral brain regions. Representative images showing α-syn inclusions in the ipsilateral FrC, Ctx, Hypo-thal, BS, and Cere of rats following intrastriatal injection of intestine or vagus nerve lysate derived from a post-mortem PD patient. No obvious α-syn pathology was observed in the rats injected with PBS. Scale bars, 20 μm. BS, brain stem; Cere, cerebellum; Ctx, somatosensory cortex; FrC, frontal cortex; Hypo-thal, hypothalamus.

**Figure 6.** The α-syn aggregates in the intestine or vagus lysate derived from a PD patient induce microglia activation and astrogliosis in the SNc. Immunofluorescence is depicted for Iba1^+^ microglia (**a**) and GFAP^+^ astrocyte (**b**) in the ipsilateral and contralateral SNc of intestine or vagus lysate-injected rats. Scale bars, 25 μm. The ratios of reactive Iba1^+^ and GFAP^+^ cells are quantified in the SNc of intestine or vagus lysate-injected rats. Unpaired t-test. Iba1, * p = 0.015 (Intestine), * p = 0.0443 (Vagus); GFAP, *** p = 0.0009 (Intestine), * p = 0.0403 (Vagus). GFAP, glial fibrillary acidic protein; Iba1, ionized calcium-binding adaptor molecule 1; L, ipsilateral left brain; R, contralateral right brain.
